# Comparison of bilateral implantation of monofocal intraocular lenses with enhanced intermediate function targeting with − 2.00 D and emmetropia in moderate to high myopic Asian patients

**DOI:** 10.1186/s40662-024-00410-4

**Published:** 2024-11-01

**Authors:** Yoo Young Jeon, Hayoung Lee, Kyu Sang Eah, Nahyun Park, Ho Seok Chung, Jae Yong Kim, Hungwon Tchah, Hun Lee

**Affiliations:** 1 Department of Ophthalmology, Asan Medical Center, University of Ulsan College of Medicine, 88, Olympic-Ro 43-Gil, Songpa-Gu, Seoul, 05505 South Korea; 2 Department of Ophthalmology, Kim’s Eye Hospital, Myung-Gok Eye Research Institute, Konyang University College of Medicine, Seoul, South Korea; 3 Department of Ophthalmology, Brain Korea 21 project, University of Ulsan College of Medicine, Seoul, South Korea; 4 Center for Cell Therapy, Asan Medical Center, Seoul, South Korea

**Keywords:** Myopia, Cataract surgery, Monofocal intraocular lenses, Intermediate vision

## Abstract

**Background:**

To investigate the outcomes of bilateral implantation of enhanced monofocal intraocular lenses (IOLs, ICB00) with a − 2.00 diopter (D) target in patients with moderate to high myopia and to compare the clinical outcomes of a − 2.00 D binocular target with an emmetropia target in patients who underwent cataract surgery.

**Methods:**

In this retrospective study, we reviewed the medical records of patients who underwent uncomplicated phacoemulsification with ICB00 IOL implantation. Emmetropia (Group 1) and − 2.00 D (Group 2) were targeted in 60 and 20 eyes of 30 and 10 patients, respectively. Three months after surgery, uncorrected distance visual acuity (UDVA), corrected distance visual acuity (CDVA), uncorrected intermediate visual acuity (UIVA), and uncorrected near visual acuity (UNVA) were measured. Defocus curves were measured under the photopic condition by intervals of 0.50 D from + 0.50 D to − 4.00 D.

**Results:**

The postoperative binocular logMAR UDVA, UIVA, and UNVA were 0.01 ± 0.03, 0.08 ± 0.11, and 0.33 ± 0.15 in Group 1 and 0.31 ± 0.13, 0.04 ± 0.05, and 0.11 ± 0.07 in Group 2, respectively. Group 2 showed a significantly superior postoperative binocular UNVA (*P* = 0.027) and inferior binocular UDVA (*P* = 0.003) than Group 1. Binocular UIVA and CDVA did not significantly differ between the groups although UIVA was better in Group 2 than in Group 1. Near glasses were needed by 66% of Group 1 and 0% of Group 2.

**Conclusions:**

Bilateral implantation of ICB00 IOL with − 2.00 D of residual myopia is suitable for patients with moderate to high myopia to improve UDVA, UIVA, and UNVA.

## Background

Recently, patients are increasingly desiring both near and far vision after cataract surgery. Since the glasses-free lifestyle is increasingly popular and technology has improved to meet the demand, multifocal intraocular lens (IOL) implantation has become an effective way to correct presbyopia in patients who undergo cataract surgery [1]. Similarly, various IOLs, such as extended depth-of-focus (EDoF), bifocal, and trifocal IOLs, have been developed and introduced.

The monofocal IOL with enhanced intermediate function (Eyhance, Tecnis ICB00, Johnson & Johnson Vision Care Inc, Jacksonville, FL, USA) is a new monofocal refractive lens that can better enhance intermediate visual acuity compared with monofocal IOLs. It has the same features as the ZCB00 IOL, except for the modified aspheric anterior surface of the optic. The Eyhance monofocal IOL shows no difference in distance visual acuity and is significantly better in improving intermediate visual acuity compared with monofocal IOLs, making it useful for patients who want to see at intermediate distances without glasses [2]. Redruello-Guerrero et al. [3] reported that the postoperative intermediate visual acuity was significantly higher with the Eyhance monofocal IOL than with the ZCB00 monofocal IOL. In addition, glare and halo, which are the chief disadvantages of multifocal IOLs, can be reduced while providing good far and intermediate vision [1, 2]. Patients have been increasingly satisfied with this monofocal IOL with enhanced intermediate function when targeting emmetropia because it provides an extended range of vision [4, 5].

However, a substantial proportion of the Asian population has moderate to high myopia. These patients could previously read text on a smartphone or simple documents without glasses. However, after cataract surgery targeting emmetropia, such activities became impossible, resulting in a significant lifestyle change. Patients with myopia have often adjusted to their near vision without glasses before cataract surgery; therefore, only targeting far distances can cause them to become unable to see small letters using near vision, which can be a source of dissatisfaction after cataract surgery [6]. Kora et al. [7] found that, in 121 patients with high myopia who underwent cataract surgery, most patients preferred 0.00 diopter (D) and − 3.00 D corrections after cataract surgery. Therefore, many patients with myopia have performed cataract extraction with implantation of a monofocal IOL, resulting in a spherical equivalent (SE) of approximately − 3.00 D for near vision [7, 8].

Regarding monofocal IOL with enhanced intermediate function implantation in patients with myopia, a previous study of 112 eyes with a non-dominant eye target of − 0.75 D showed a low rate of photic phenomena (4%–13%), with 92% of patients being satisfied with their IOL selection [4]. However, there have been no reports evaluating the clinical outcome and patient satisfaction with a myopic target of − 2.00 D or more in patients with moderate to high myopia with a monofocal IOL with enhanced intermediate function. Therefore, in this study, we aimed to investigate the clinical outcomes of cataract surgery with a target of − 2.00 D bilaterally using monofocal IOLs with enhanced intermediate function in patients with cataracts with moderate to high myopia.

## Methods

In this retrospective, observational cohort study, we reviewed the medical records of patients who underwent uncomplicated phacoemulsification with implantation of monofocal IOLs with enhanced intermediate function between February 2020 and October 2022 in the Department of Ophthalmology, Asan Medical Center. This study was reviewed and approved by the Institutional Review Board of the Asan Medical Center and University of Ulsan College of Medicine (IRB No. 2024−0539) and conducted in accordance with the tenets of the Declaration of Helsinki. This study has been reported following the STROBE guidelines.

Overall, 80 eyes of 40 patients were enrolled. All patients had pre-existing moderate to high myopia, 60 eyes of 30 patients had a target of emmetropia (Group 1) and 20 eyes of 10 patients had a target of − 2.00 D (Group 2). The exclusion criteria were as follows: (1) previous ocular trauma or ocular surgery, including corneal, refractive, or retinal surgery; (2) corneal disease including infection, opacity, and irregularities; (3) other ocular diseases that can affect vision including glaucoma, age-related macular degeneration, diabetic retinopathy, and retinal vascular occlusions; (4) using systemic or ocular medications that affect vision; and (5) complications during cataract surgery.

All patients underwent preoperative and 3-month postoperative ophthalmologic examinations. Visual acuity was measured using the Snellen chart and converted to logMAR. Uncorrected distance visual acuity (UDVA) and corrected distance visual acuity (CDVA) assessment, slit-lamp examination, auto-refraction and auto-keratometry (Canon R-50, Canon USA Inc., Huntington, NY, USA), corneal topography (Orbscan, Bausch & Lomb, Rochester, NY, USA), axial length (AL) measurement (IOL Master 700, Carl Zeiss Meditec, Jena, Germany), and fundus examinations were completed preoperatively. Three months after surgery, UDVA, CDVA, uncorrected intermediate visual acuity (UIVA) at 66 cm, uncorrected near visual acuity (UNVA) at 33 cm, auto-refraction, and auto-keratometry were measured. Monocular and binocular defocus curves were measured under photopic conditions by intervals of spherical 0.50 D from + 0.50 D to − 4.00 D. At 3 months after surgery, all patients were surveyed regarding their overall satisfaction and need for near glasses. Overall satisfaction was rated on a 5-point scale as follows: 1 = very dissatisfied, 2 = dissatisfied, 3 = neither satisfied nor dissatisfied, 4 = satisfied, and 5 = very satisfied. We also scored the occurrence of glare and halos on a 5-point scale as follows: 1 = not at all, 2 = rarely, 3 = occasionally, 4 = often, and 5 = always. The scale used to assess satisfaction has not been validated, and we used a questionnaire created specifically for this study.

### Surgical technique

During the cataract surgery, the Catalys femtosecond laser platform (Johnson & Johnson Vision Care, Inc) was used for all patients. Continuous curvilinear capsulorhexis and lens fragmentation were performed. Crystalline lens fragmentation was performed using a standard template with a pattern described as “lens softening: quadrants” in the system. A clear corneal incision of 2.2 mm was made. Hydrodissection and phacoemulsification were performed using Signature Pro (Johnson & Johnson Vision Care, Inc). The monofocal IOL with enhanced intermediate function was then implanted into the capsular bag, and all corneal incisions were sealed through stromal hydration. Postoperative topical medications included levofloxacin hydrate 1.5% (Cravit 1.5%, Santen Pharmaceutical, Osaka, Japan) four times daily, prednisolone acetate 1.0% (Predforte 1.0%, Allergan, Inc.) four times daily, ketorolac tromethamine 0.45% (Acuvail, Allergan, Inc.) two times daily, and cyclosporin 0.1% (Ikervis 0.1%, Santen Pharmaceutical) once daily.

### Statistical analysis

Statistical analysis was performed using SPSS for Windows statistical software (v.25.0; SPSS Inc., Chicago, IL, USA). The normality of the data was analyzed using the Shapiro–Wilk test. The differences in preoperative data and postoperative outcomes between the two groups were compared using the Mann–Whitney U test.

## Results

Of the 80 eyes, 60 were included in Group 1 (emmetropic target) and 20 in Group 2 (myopic target). Table 1 shows the baseline demographics and preoperative parameters of both groups. The mean age of the patients was 70.47 ± 8.02 years (53 to 80 years) in Group 1 and 64.90 ± 5.08 years (59 to 75 years) in Group 2, with no significant difference between the two groups (*P* = 0.165). The preoperative SE was − 0.12 ± 1.48 D (− 4.88 to 2.25 D) in Group 1 and − 5.23 ± 3.86 D (− 11.63 to − 1.75 D) in Group 2, with a significant difference between the two groups (*P* < 0.001). The AL was 23.52 ± 1.09 mm (21.38 to 25.98 mm) in Group 1 and 25.62 ± 1.00 mm (24.3 to 28.02 mm) in Group 2 (*P* = 0.087, Table 1).

**Table 1:** Demographics and preoperative parameters of the patients

Parameter	Emmetropic target (Group 1)	Myopic target (− 2.00 D) (Group 2)	*P* value*	
Age (years)	70.47 ± 8.02 (53–80)	64.90 ± 5.08 (59–75)	0.165	
Preop monocular CDVA (logMAR)	0.22 ± 0.25 (0.00–1.00)	0.28 ± 0.23 (0.22–1.15)	0.731	
Preop SE (D)	− 0.12 ± 1.48 (− 4.88 to 2.25)	−5.23 ± 3.86 (− 11.63 to −1.75)	< 0.001	
Corneal astigmatism (D)	0.66 ± 0.44 (0.00–1.50)	0.75 ± 0.40 (0.00–1.25)	0.825	
Axial length (mm)	23.52 ± 1.09 (21.38–25.98)	25.62 ± 1.00 (24.3–28.02)	0.087	

*D* = diopter; *logMAR* = logarithm of the minimal angle of resolution; *preop* = preoperative; *CDVA* = corrected distance visual acuity; *SE* = spherical equivalent

*Statistical analysis was performed with Mann-Whitney U test. Results reported as mean ± SD

**Fig. 1:**
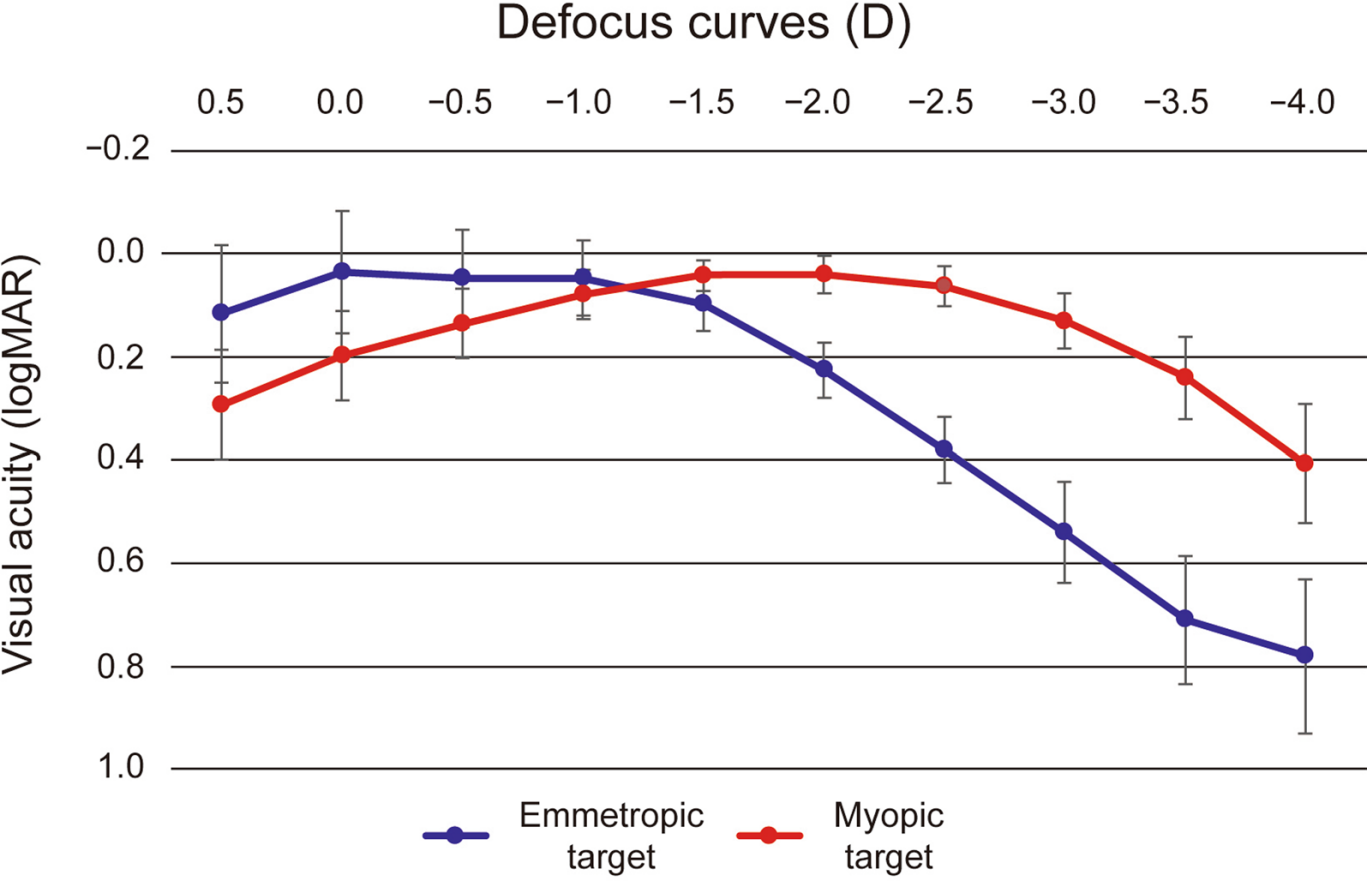
Mean binocular defocus curves between the emmetropic and myopic target groups. D, diopter

The postoperative SE was − 0.03 ± 0.42 D (− 0.75 to 0.63 D) in Group 1 and − 2.16 ± 0.53 D (− 3.63 to − 1.75 D) in Group 2, indicating that postoperative myopia was statistically higher in Group 2 (*P* < 0.001) (Table 2). Figure 1 shows the results of the postoperative defocus curves of both groups. Group 1 had visual acuity better than 20/30 between 0.00 D and − 2.00 D in binocular vision, and Group 2 had visual acuity better than 20/30 between − 0.50 D and − 3.00 D in binocular vision, with relatively good visual acuity in the distance from 30 cm to 3 m.

**Table 2:** Comparison of visual outcomes between the two groups at 3 months postoperatively

Binocular VA	Emmetropic target (Group 1)	Myopic target (− 2.00 D) (Group 2)	*P* value*
UDVA (logMAR)	0.01 ± 0.03	0.31 ± 0.13	0.027
UIVA (logMAR)	0.08 ± 0.11	0.04 ± 0.05	0.593
UNVA (logMAR)	0.33 ± 0.15	0.11 ± 0.07	0.003
CDVA (logMAR)	0.01 ± 0.03	0.02 ± 0.03	0.160
Postop SE (D)	−0.03 ± 0.42 (− 0.75–0.63)	−2.16 ± 0.53 (− 3.63 – −1.75)	< 0.001

*VA* = visual acuity; *logMAR* = logarithm of the minimal angle of resolution; *D* = diopter; *UDVA* = uncorrected distance visual acuity; *UIVA* = uncorrected intermediate visual acuity; *UNVA* = uncorrected near visual acuity; *CDVA* = corrected distance visual acuity; *postop* = postoperative; *SE* = spherical equivalent

*Statistical analysis was performed with Mann-Whitney U test. Results reported as mean ± SD

The postoperative binocular logMAR UDVA, UIVA, and UNVA were 0.01 ± 0.03, 0.08 ± 0.11, and 0.33 ± 0.15 in Group 1 and 0.31 ± 0.13, 0.04 ± 0.05, and 0.11 ± 0.07, in Group 2, respectively (Table 2). The postoperative binocular UDVA was significantly better in Group 1 than in Group 2 (*P* = 0.027). Considering that Group 2 patients had a target of myopia of − 2.00 D, we had predicted that the postoperative binocular UDVA in Group 1 would be superior to that in Group 2; however, the CDVA was not different between the two groups. In terms of postoperative binocular UNVA, Group 2 showed significantly better visual acuity than Group 1 (*P* = 0.003). Binocular UIVA was not different between the two groups, although the UIVA in Group 2 tended to be better than that in Group 1.

**Fig. 2:**
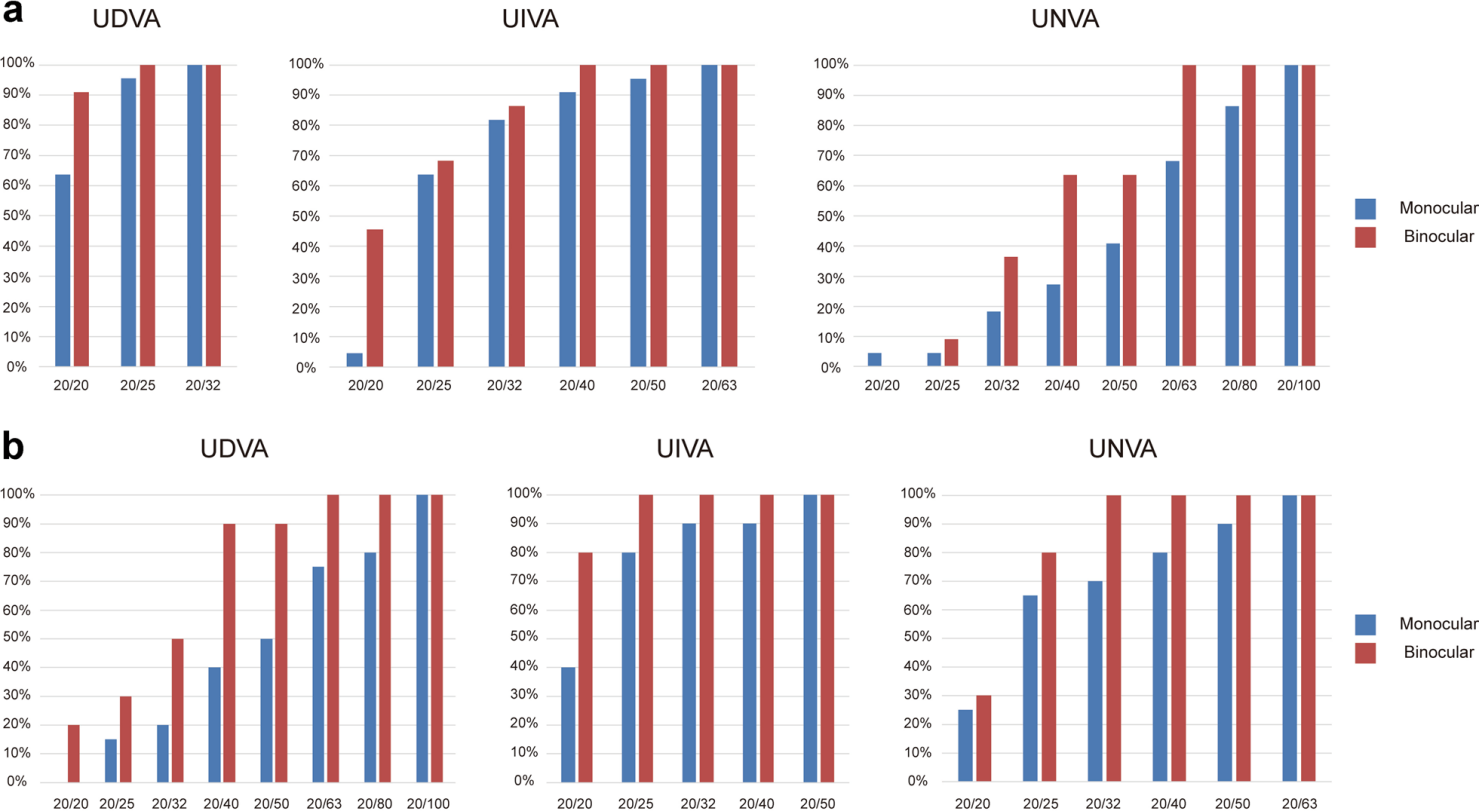
Postoperative 3 months visual outcomes of emmetropic (**a**) and myopic (**b**) target group. UDVA, uncorrected distance visual acuity; UIVA, uncorrected intermediate visual acuity; UNVA, uncorrected near visual acuity

At 3 months postoperatively, 100% of patients in Group 1 had a binocular UDVA of 20/25 or better. Additionally, 100% of patients had a binocular UIVA of 20/40 or better and 86% had a binocular UIVA of 20/32 or better in Group 1 (Fig. 2a). All (100%) patients in Group 2 showed a binocular UIVA of 20/25 or better. Additionally, 80% of patients showed a binocular UNVA of over 20/25, and 100% showed a binocular UNVA of over 20/32 in Group 2. Furthermore, 90% of patients showed a binocular UDVA over 20/40 in Group 2 (Fig. 2b).

**Fig. 3:**
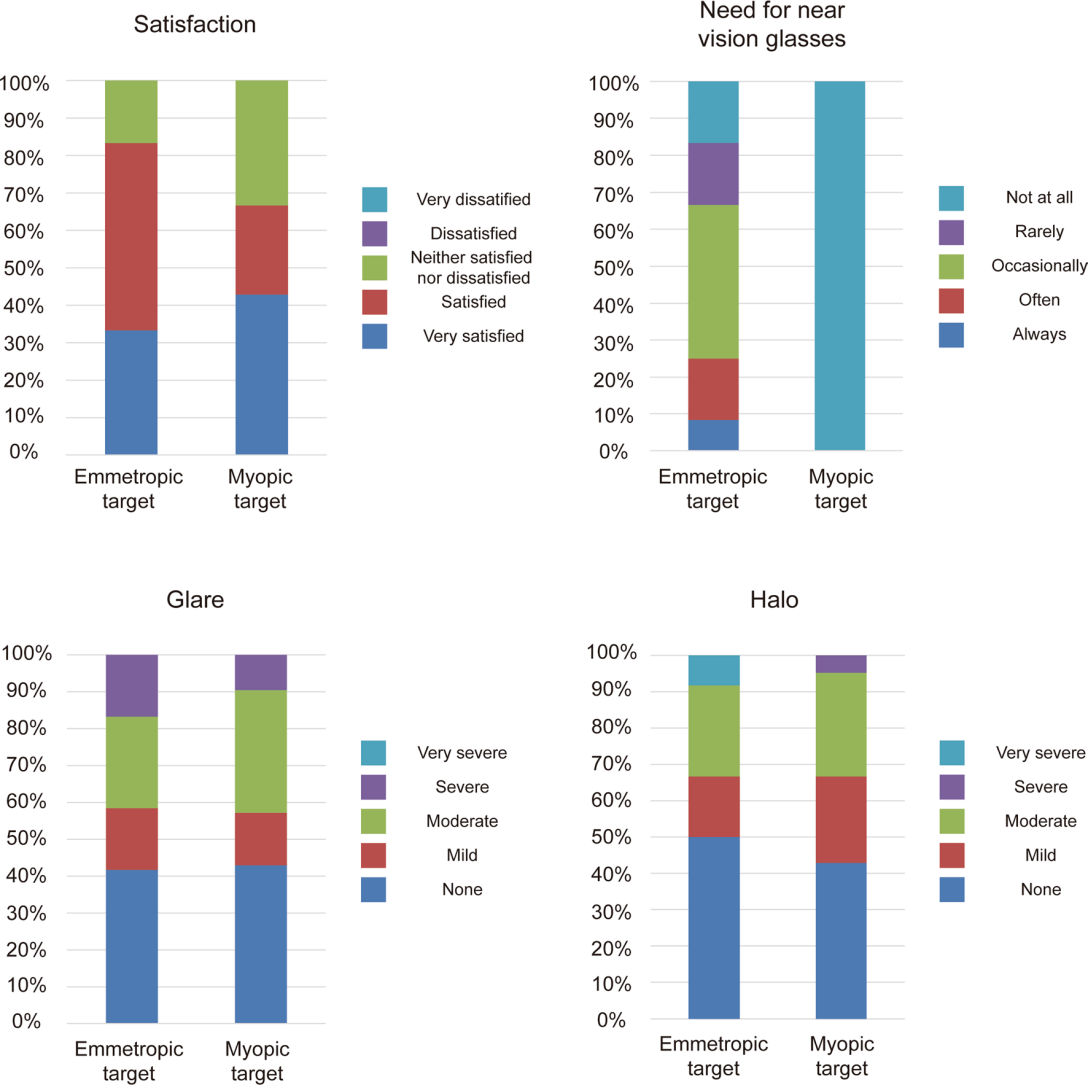
Comparison of patients’ subjective satisfaction, need for near vision glasses, photic phenomenon (glare and halo) between the emmetropic and myopic target groups

In terms of subjective satisfaction, no patient in either group selected “dissatisfied” or “very dissatisfied,” and the percentages of “very satisfied” and “satisfied” responses were higher in Group 1, with more “neither satisfied nor dissatisfied” responses in Group 2 (Fig. 3). Near glasses were not needed by 100% of the patients in Group 2, compared with 34% of patients in Group 1 (Fig. 3). Regarding glare and halo, 17% of patients complained of severe glare in Group 1 and 10% in Group 2. A total of 8% of patients complained of very severe halo in Group 1 and 4% complained severe halo in Group 2. Overall, neither group exhibited severe glare and halo, and there was no significant difference between the groups (*P* = 0.841, Fig. 3).

No intraoperative or postoperative complications of cataract surgery, such as corneal dysfunction, cystoid macular edema, endophthalmitis, secondary glaucoma, posterior capsular rupture, rhegmatogenous retinal detachment, or IOL dislocation or subluxation, were noted. None of the patients underwent secondary surgery.

## Discussion

Herein, we investigated the clinical outcomes and patient satisfaction after implantation of monofocal IOLs with enhanced intermediate function in patients with moderate to high myopia, targeting mild myopia of − 2.00 D instead of emmetropia. There was no difference in CDVA between patients targeting postoperative myopia of − 2.00 D and those targeting emmetropia. However, patients targeting postoperative myopia of − 2.00 D achieved significantly better UNVA than those targeting emmetropia. The UIVA in Group 2 tended to be better than that in Group 1. Photic phenomena such as glare and halo did not differ between the two groups, with the incidence being relatively low in both groups.

The ICB00 IOL can be used to correct presbyopia when multifocal IOLs are not suitable since it can provide better intermediate and near vision compared with monofocal IOLs [9–11]. A study by Mencucci et al. [2] comparing the outcomes of the ZCB00 and ICB00 IOLs in 80 eyes found that the ICB00 provided relatively good intermediate visual acuity, which reduced the patient’s dependence on glasses. Koh et al. [12] also found similar results, in that the ICB00 group showed significantly better UIVA and UNVA than the ZCB00 group at 3 months postoperatively. Cinar et al. [13] found no difference in CDVA, UDVA, CNVA, and UNVA between the ICB00 IOL and another monofocal IOL (SN60WF IOL), while CIVA and UIVA were significantly better in patients with the ICB00 IOL. A study conducted by Corbelli et al. [14] comparing the clinical outcomes of the ICB00 IOL with those from the EDoF IOL (Symfony ZXR00) reported that the ICB00 IOL provided binocular UIVA similar to that of the ZXR00, with a comparable spectacle independence score between the two IOLs. While the contrast sensitivity score was similar between the two IOLs, photic phenomena such as halo and glare were more severe in eyes with ZXR00 IOL. Another recent study showed that monocular and binocular UDVA, UIVA, and CDVA were similar between the 48 eyes implanted with ICB00 IOLs bilaterally and 40 eyes with EDoF ZXR00 IOL implantation. Monocular UNVA and spectacle independence for near distance were better in the ZXR00 group, whereas binocular UNVA did not differ significantly between the two IOLs [15].

Considering these advantages, the ICB00 IOL can be a good option for patients not pursuing a multifocal or trifocal IOL who need near or intermediate vision [16–18]. Therefore, we assumed that the benefits of bilateral ICB00 IOL implantation in patients with moderate to high myopia, targeting mild myopia of − 2.00 D, can maintain their existing near vision and improve the UDVA after cataract surgery. A recent study comparing the results of ICB00 IOLs targeting mild monovision (nondominant eye target of − 0.75 D) with those targeting emmetropia in both eyes showed that the UIVA and UNVA was one line better in the monovision group without any difference in UDVA. Additionally, the monovision group tended to have less difficulty and higher satisfaction with near and intermediate vision [19]. In patients with myopia who previously had relatively good near vision, achieving emmetropia after cataract surgery may cause severe complaints. Jaafar et al. [20] studied 139 patients after cataract surgery, dividing them into two groups: patients with a target of emmetropia and those with a target of residual myopia (− 0.50 D to − 1.00 D), and examined the effects of postoperative near vision satisfaction and quality of life. The results showed that overall satisfaction did not differ between the two groups, but near vision satisfaction was significantly higher in the residual myopia group, especially for reading small print letters (font sizes 8 to 9). Similarly, patients with myopia who are used to near vision without glasses may have less postoperative satisfaction with surgery targeting emmetropia. In patients with myopia, targeting emmetropia inevitably renders their intermediate and near vision even worse than that before the cataract surgery. Hayashi et al. [6] previously studied the optimal target for cataract surgery in patients with myopia who were operated on by targeting − 1.00, − 1.50, − 2.00, − 2.50, or − 3.00 D. Patients targeting postoperative myopia of − 2.00 D showed visual acuity better than 20/30 at distances of 0.7, 0.5, and 0.3 m, suggesting that postoperative myopia of − 2.00 D could be an optimal target for patients with pre-existing mild myopia.

Trifocal IOLs can be another good option for patients with pre-existing myopia, allowing them to see near and far distances without glasses. Kim et al. [21] investigated postoperative near vision spectacle independence in patients with mix-and-match implantation of the EDoF (AT LARA 829MP) and trifocal IOLs (AT LISA tri839MP). Of these, 79.4% did not require reading glasses, but 20.6% did, and near vision spectacle independence was lower in patients with more preoperative myopia. Thus, they found that preoperatively, patients with myopia who underwent mix-and-match implantation tended to require glasses for near vision. They recommended that surgeons should consider this before surgery, as patients with myopia have significant difficulty achieving near vision with trifocal IOLs. However, in our study, we achieved better results than this mix-match implantation as 100% of the patients implanted with ICB00 IOLs targeting myopia did not require near glasses.

Other studies have compared trifocal IOL outcomes in patients with high and extremely high myopia to those in patients with normal AL. Meng et al. [22] compared the outcomes of a trifocal IOL (AT LISA, tri 839MP) in the control (AL < 26 mm), high myopia (AL 26 to 28 mm), and extreme myopia groups (AL ≥ 28 mm). In control and high myopia groups, approximately 60% of eyes achieved UNVA and UIVA of 0.10 logMAR or better, but significantly fewer eyes in the extreme myopia group achieved 0.10 logMAR or better. Defocus curves revealed that the visual acuity was significantly worse in the extreme myopia group than in the other groups at 0.00, − 0.50, and − 2.00 D. These results suggest that surgeons should consider poor satisfaction with trifocal IOLs in patients with high myopia and extremely high myopia.

No patient in either group indicated that they were dissatisfied or very dissatisfied, and the percentage of patients who were very satisfied or satisfied was higher in patients targeting emmetropia. The relatively lower satisfaction rate with the ICB00 IOL with a − 2.00 D target than the emmetropic target was attributed to the inability to meet the postoperative expectations of UDVA in patients with myopia. Previous studies have shown that the incidence of posterior subcapsular cataracts is significantly higher in patients with myopia, and they suffer from cataracts at earlier ages compared with patients without myopia, resulting in a severe reduction in visual acuity and quality of life [22]. Accordingly, patients with high myopia tend to have cataract surgery at a younger age than patients without myopia and have higher expectations of postoperative improvement in visual function and quality of life [24]. Therefore, bilateral implantation of monofocal IOL with enhanced intermediate function in patients with moderate to high myopia, targeting mild myopia of − 2.00 D, could result in a relatively lower satisfaction score than expected because of decreased UDVA. Hence, preoperative education in younger patients with cataracts with moderate to high myopia is crucial for avoiding disappointment.

Reduction in photic phenomena, such as glare and halos, which caused patient dissatisfaction with trifocal IOLs, is a relative advantage when using the ICB00 IOL. Several studies have shown that the ICB00 IOL has better contrast sensitivity than trifocal IOLs, and multifocal IOLs have limitations with unwanted effects such as halo and glare [18, 25–27]. Except for the high-order aspheric central zone, the physical structure of the ICB00 IOL closely resembles that of its monofocal counterpart; thus, contrast sensitivity is not compromised for improving the depth of focus [28]. We found similar results, in that patients with bilateral ICB00 IOL implantation experienced less discomfort, with lower percentages of glare and halos in the emmetropia and myopic targeted groups.

This study has several limitations. First, the sample size of the group with a residual myopia target of − 2.00 D with the Eyhance IOL was small. A long-term prospective study with a larger sample size is required. Second, the randomization was not perfectly performed because the patients decided, during preoperative counseling, whether they wished to use glasses or desired near vision without glasses. Third, we did not evaluate variables such as the patients’ underlying diseases; therefore, we cannot confirm their impact. Lastly, targeting bilateral myopia can be inconvenient because it might require the use of glasses during all daily tasks, and micro-monovision would therefore be a good alternative.

## Conclusions

We demonstrated that in patients with moderate to high myopia, bilateral implantation of monofocal IOLs with enhanced intermediate function targeting with − 2.00 D is a suitable option to achieve satisfactory UDVA, UIVA, and UNVA. Therefore, surgeons may consider the bilateral implantation of ICB00 IOLs targeting with − 2.00 D for patients with cataract who have moderate to high myopia to achieve satisfactory visual acuity at various distances without changing the patient’s lifestyle and causing severe photic phenomenon.

## Data Availability

The data will be made available on request.
